# Use of Encapsulated and Raw Organic Acids for Inhibition of *Salmonella* in a Refrigerated Raw Meat-Based Diet (RMBD) Matrix for Dogs

**DOI:** 10.3390/ani16203232

**Published:** 2026-10-14

**Authors:** Samuel Kiprotich, Jared Lou Rivera, Shivaprasad Doddabematti Prakash, Larissa Alves Koulicoff, Robert Mason, Eric Altom, Julia Guazzelli Pezzali, Kaliramesh Siliveru, Charles Gregory Aldrich

**Affiliations:** 1 General Mills, Wilton, CT 06897, USA; 2 Department of Grain Science and Industry, Kansas State University, Manhattan, KS 66506, USA; jdrivera07@ksu.edu (J.L.R.);; 3 Animal Nutrition & Health Division, Balchem Corporation, Montvale, NJ 07645, USA; 4 Nulo Pet Food, Austin, TX 78746, USA

**Keywords:** organic acids, encapsulation, food safety, raw diets, pet food

## Abstract

The rising demand for using raw meat diets (RMBDs) for feeding companion animals requires improvement in their food safety. Organic acids could be a promising anti-microbial ingredient for mitigating pathogen contamination. This study investigated the effects of encapsulated (E) and raw (R) acids (citric—CE and CR; lactic—LE and LR) on *Salmonella* survival in RMBDs during refrigerated storage. The acidulants (1.0% LE, 1.0% CE, 0.5% LE + 0.5% CE, 0.5% CE + 0.5% LR, 0.5% LE + 0.5% CR, and 0.5% LR + 0.5% CR) were incorporated in RMBD patties inoculated with *Salmonella* (ATCC 8326, ATCC 14802, and ATCC 13076) at 6 and 9 log CFU/patty levels. Inoculated (PC) and non-inoculated (NC) patties made without acidulants were used as controls. Patties were stored at 4 °C and *Salmonella* enumeration was done at days 0, 1, 4, 7, 10, 13, 16, 19, and 22. *Salmonella* reduction was higher (*p* < 0.05) in stored patties with both encapsulated and raw acids (3.10–4.10 logs) added relative to using individual encapsulated acids (2.40–3.70 logs) and control (0.70–0.90 logs). Higher inoculation levels resulted in greater (*p* < 0.05) *Salmonella* reductions in the patties after storage. The reductions were influenced (*p* < 0.05) by the treatment × time interaction with longer storage time resulting in higher reductions. The *Salmonella* inactivation under all acid treatments followed the Weibull model (R^2^—0.86 to 0.98; RMSE—0.19 to 0.34; β—1.23 to 2.08). The results show that encapsulated organic acids can inhibit *Salmonella* growth and survival in RMBDs under refrigerated conditions. The results from this study could be used as basis for improving the food safety of RMBDs.

## Simple Summary

*Salmonella* contamination in raw pet food is a major concern due to the risk of causing illness to pets and people who handle the food. This study tested whether organic acids could reduce *Salmonella* in raw dog food kept under refrigeration. The addition of these acids reduced the *Salmonella* present in the raw dog food during refrigerated storage. The findings suggest that organic acids could be a viable ingredient for improving the safety of raw pet foods against *Salmonella* contamination.

## 1. Introduction

The commercial pet food space is dominated by ultra-processed diets (UPCDs) produced with thermal process steps such as extrusion, canning, and baking [1,2]. However, these processes result in the formation and consumption of advanced glycation products (e.g., acrylamide), fat oxidation, and altered digestibility and nutrient availability in the finished product [3,4]. These events have led to consumers’ concerns on the long-term companion animal health impacts from the frequent consumption of UCPDs, which have resulted in an increased demand for minimally processed pet foods. 

Minimally processed pet foods include semi-moist, raw meat-based (RMBD), biologically appropriate pet food (BARFs), and raw animal (RAP) pet food categories; these products differ from UCPDs as these are processed with minimal to no thermal process steps [5]. The category of RMBDs is considered a fast-growing market segment, with a 2024 market size of $18.2 billion and a compound annual growth rate (CAGR) of 6.5% [6]. RMBDs are defined as pet foods that may not be thermally processed (e.g., retort, cooking, etc.), rendered, hydrolyzed or fermented [7]. Hence, its production is based on using uncooked animal-based proteins fortified with ingredients. However, this comes with downsides such as having incomplete nutrient profiles, improper mineral ratios, and lack of essential micronutrients for the companion animal. To offset these limitations, RMBDs are often formulated with the inclusion of other ingredients such as tubers, vegetables, fruits, and cereals to make it more nutritionally balanced [5,8].

The minimal production process of RMBDs aids the increased pet owner perception of RMBDs being a more nutritious, healthier, and natural option for their pets [5,9]. However, these production practices compromise the food safety of RMBDs as raw animal-based proteins (e.g., beef, chicken, etc.) are historically known to be potentially contaminated with enteric pathogens. Solis et al. [10] reported that 35.7% (n = 42) of RMBDs were positive for *Salmonella* and *Listeria monocytogenes*; these pathogens were also isolated from the fecal matter of RMBD-fed dogs, while extruded diet-fed dogs produced fecal matter without pathogenic microorganisms. Furthermore, dogs fed with RMBDs are reported to be more prone to testing positive for *Salmonella* and multi-drug resistant (MDR) *E. coli* variants [11,12]. These studies highlight the public health significance of RMBDs as they could facilitate the transmission of foodborne illnesses to both humans and pets. Morelli et al. [13] reported that 28% (n = 218) of dog owners were living with immunocompromised individuals who are at increased risk of succumbing to enteric foodborne illnesses transmitted by pathogens from the diet of their companion animals. Empert-Gallegos et al. [14] reported that 20% (n = 419) of dog owners who fed their dogs with RMBDs regarded them as potential vectors for foodborne pathogen transmission. Lack of consumer awareness on the public health risk of RMBDs is also a major factor. Viegas et al. [12] reported that 87.9% and 98.8% of their survey respondents (n = 412) in Brazil believed that RMBDs do not pose any health hazards to animals or humans.

The higher risk of pathogen contamination in RMBDs requires the need for developing pathogen reduction strategies suitable for their production process. Aside from its public health significance, the development of such strategies is also needed to comply with current animal food safety regulations. For instance, the 2017 Food Safety and Modernization Act requires animal food processing facilities to implement risk-based preventive controls to mitigate biological, chemical, and physical hazards that could occur in animal food production [15]. High-pressure processing (HPP) is a common process control step used for pathogen reduction in RMBDs; however, its downsides of high capital cost, complex processing requirements, and potential for increasing pathogen resistance in the food matrix prompt the need for more accessible alternatives [5,16,17].

Organic acids, such as citric and lactic acid, are compelling alternatives as a pathogen reduction measure as they could be uniformly distributed within the RMBD matrix due to the higher surface area caused by the grinding step. This potentially allows better interaction between the acidulants and the pathogens potentially present in RMBDs resulting in better reductions. For instance, Kiprotich et al. [1] reported a ≈ 2.0 to 3.0 log reduction in *Salmonella* in stored RMBDs treated with encapsulated and raw acids (citric and lactic) at 1.0 to 3.0% *w*/*w* application rate. However, higher doses caused quality defects such as color degradation, poorer texture, syneresis, and weeping. In addition, organic acids have been used in food production for a long time, present in a lot of common foods, and are likely familiar to pet owners. These characteristics make organic acids a suitable anti-microbial ingredient for RMBDs’ natural and healthier perception to pet owners. In addition, these ingredients could be a more accessible alternative for mitigating *Salmonella* contamination in RMBDs.

Despite the anti-microbial effectiveness of organic acids demonstrated, limited information is available on their efficacy when applied in different forms (e.g., encapsulated vs. raw) in practical scenarios in RMBD manufacturing. Furthermore, additional research is needed to determine effective acidulant treatments that can achieve meaningful *Salmonella* reductions while minimizing undesirable quality changes to RMBDs. This study hypothesized that using encapsulated acids, either alone or combined with raw acids, can be an effective intervention for reducing *Salmonella* contamination in refrigerated RMBDs. This study also hypothesized that the usage of encapsulated acids could result in an improved anti-microbial performance compared to raw organic acids. The objectives for this study were to explore the efficacy of different encapsulated and raw acids against *Salmonella* and characterize its inactivation kinetics in RMBDs during a 22-day refrigerated storage study. The results from this study can be used as basis for further developing organic acid-based mitigation strategies for pathogen contamination in RMBD production.

## 2. Materials and Methods

### 2.1. Salmonella Serovars and Preparation of Working Inoculum

*Salmonella enterica* serovars including Typhimurium (ATCC 14802), Enteritidis (ATCC 13076), and Heidelberg (ATCC 8326) were procured from the culture collection of the Feed Toxicology and Microbiology Laboratory at Kansas State University (Manhattan, KS, USA). The cultures were frozen (−80 °C) in cryogenic vials in a 7:3 mixture ratio of Tryptic Soy Broth (TSB) (Difco; Becton, Dickinson and Company, Sparks, MD, USA) and glycerol. The vials were thawed, and their contents were transferred to fresh TSB (10 mL) followed by incubation at 37 °C for 24 h. The working cultures were prepared via 2 consecutive, 24 h transfers of the activated cultures into TSB (100 mL), followed by incubation at 37 °C. The incubated TSB suspension (30 mL/serovar) was centrifuged (5000× *g*, 10 min, 20 °C) using a Sorvall X1R centrifuge (Thermo Scientific, Waltham, MA, USA). The cells from each serovar were then suspended in 3.0 mL of 0.85% *w*/*v* NaCl and combined in equal volumes to obtain a final viable cell concentration of ≈10.00 log CFU/ml (plated in TSA) that was used directly or diluted further to ≈7.00 log CFU/ml (plated in TSA) using 0.85% *w*/*v* NaCl.

### 2.2. Ingredients, Preparation and Inoculation of RMBD Patties

The RMBD model was prepared under sanitary conditions in the Pet Food Processing Lab (Manhattan, KS, USA). Human-grade ingredients including turkey, sweet potato, chicken liver, carrots, and apples were purchased from a local grocery store (Manhattan, KS, USA). The Petshure^TM^ encapsulated and dry-plated (raw) citric and lactic acids were provided by Balchem Corporation (Montvale, NJ, USA). For encapsulated acids, the encapsulation process was performed by coating the food acidulants with edible vegetable oil films to form powdery flakes with an acidulant-to-vegetable ratio of 30:70. The dry-plated/raw acidulant was coated with a filler at a 60:40 acidulant-to-filler ratio. Hence, the encapsulated and dry-plated acidulants contain 30 and 60% of acid within their matrices, respectively. The encapsulation process was done to protect the organic acids from premature reactions (e.g., neutralization) from encountering the meat matrix and providing a controlled release of the acids into the RMBD matrix.

The ingredients were weighed (2 kg batch size/treatment) according to a custom, in-house formula based on the previous study conducted by Kiprotich et al. [18]. The formulation used (%*w*/*w*) consisted of ground turkey at 75.9–83.3%, chicken liver at 4.4–5.6%, sweet potato at 4.6–6.9%, carrots at 3.7–5.8%, apples at 3.7–5.8%, and micronutrient supplement at 1.2–2.2%. The acidulant treatments (individual or combined) were weighed according to the formula ratios; the final treatment and inoculation levels used in this experiment are shown in Table 1. Both citric and lactic acids were selected as these were the more common types of acids used as food ingredients. The inclusion levels were based on a previous study by Kiprotich et al. [1,18], where these concentrations had minimal quality effects on RMBDs. The use of combined raw and encapsulated acids was done to evaluate the potential synergistic effects against *Salmonella* due to the production of a dual-release profile for the acidulants; in these treatments, the raw acidulant would dissolve immediately providing instant pH changes while the encapsulated acidulant would provide a slower release that can help maintain the pH changes against the buffering effect of the meat matrix. Negative (NC, non-inoculated) and positive (PC, inoculated) control treatments without any added acidulants were also maintained in the experiment. The ingredients were then manually mixed and shaped into 100 g patties, placed into sterile polystyrene (PS) Petri dishes (100 × 15 mm), and refrigerated (4 °C) for 2 h prior to inoculation for resting and equilibration. The meat patties formed with added acidulants typically resulted in pH values ranging from 4.5 to 5.2, while those without added acidulants had pH values ranging from 6.0 to 6.2 under similar formulations [1].

From the prepared patties under each treatment, 3 patties were randomly selected for enumeration of background *Salmonella*. For inoculation, the prepared patties (except for negative control) were removed from the refrigerator and transferred into a BSL-2 biosafety cabinet (LabConco Logic+ A2; Kansas City, MO, USA). The patties were then spot-inoculated with 0.1 mL of the prepared *Salmonella* inoculum to achieve a final concentration of ≈6.0 and 9.0 log CFU/patty (plated in XLT-4 agar). The initial inoculation levels produced by this inoculation method were verified using the same method used for enumerating background *Salmonella* in the patties. The average inoculation levels recovered from the inoculated patty samples was used as the initial *Salmonella* level for data analysis These inoculation levels were used as representative cases of the worst-case scenario of *Salmonella* contamination for this product. These levels would also ensure the presence of quantifiable levels of *Salmonella* in the patties in case large reductions occur during the duration of the refrigerated storage study due to acidulant inclusion. This allowed the proper evaluation of the microbial reductions caused by the acidulants against *Salmonella*. In addition, the use of multiple inoculation levels would allow the evaluation of inoculum effects on the efficacy of the acidulants added against *Salmonella* in RMBDs. The inoculum was gently dispersed over the patty surface using sterile L-spreaders, followed by resting (30 min) inside the biosafety cabinet to facilitate attachment and internalization of the inoculum into the patties prior to storage. This inoculation method was done to simulate *Salmonella* contamination of the patties from use of contaminated process equipment (surface contamination) and/or ingredients (internal contamination). The NC treatment, which does not contain any acidulant or inoculation, was used for testing growth of background *Salmonella* present in the patties produced. All patties were then stored in a refrigerator (4 °C) to mimic the typical storage condition used for RMBD food products. The storage study was conducted for a total of 22 days to mimic the typical shelf life of refrigerated, packaged RMBDs.

### 2.3. Salmonella Enumeration

The stored RMBD patties were sampled (1 patty per day for each treatment) at days 0, 1, 4, 7, 10, 13, 16, 19, and 22 for *Salmonella* enumeration. The patties were placed into sterile stomacher bags (710 mL; Whirl-Pak; Pleasant Prairie, WI, USA), mixed with 100 mL buffered peptone water (BPW; Difco; Becton, Dickinson and Company, Sparks, MD, USA), and homogenized using a stomacher blender (Seward; Islandia, NY, USA) for 1 min at medium speed. The homogenized suspensions were serially diluted using 0.1% peptone water (Difco; Becton, Dickinson and Company, Sparks, MD, USA), and appropriate dilutions were spread plated (0.1 mL, duplicates) on Xylose–Lysine–Tergitol-4 (XLT-4) agar plates. The inoculated plates were incubated at 37 °C for 48 h and *Salmonella* were counted as black colonies on the agar plate surface. The counts obtained were then log-transformed based on the counts and dilution plated and expressed as log CFU/patty. This plating method was also calculated to have the lowest detection limit of 2.8 log CFU/patty. The reductions were calculated as the difference in the initial *Salmonella* load of the patties at day 0 and at day t (t = 1, 4, 7, etc.).

### 2.4. Inactivation Kinetics of Salmonella During Refrigerated RMBD Storage

The *Salmonella* counts (log CFU/patty) obtained from the RMBD patties were plotted against storage time (days). The log-linear and Weibull inactivation models were fitted to describe the inactivation kinetics of *Salmonella* in treated RMBD patties during refrigerated storage. The log-linear model only describes a linear inactivation curve (i.e., similar inactivation rate across time), while the Weibull model describes a non-linear inactivation curve with varying shapes (increasing, decreasing, or constant) [19,20]. The inactivation models were fitted using the GinaFit v1.8 MS Excel Solver Add-in [21,22,23]. The equations for the fitted log-linear (Equation (1)) and Weibull (Equation (2)) models are shown below:

log(*Nt*) = log(*No*) × e^(−*kmax* × *t*)^ (1)

(2) $$\log\left(\mathrm{Nt}\right)=\log\left(\mathrm{No}\right)-{\left(\frac{t}{\delta }\right)}^{\beta }$$

For both equations, Nt and No are the populations at time t (log CFU/patty) and time = 0. For Equation (1), *kmax* is the maximum inactivation time observed (days) for the *S. enterica*, and the D-values are computed as 1/kmax when the log-linear model is used. For the Weibull model, δ is the time required for the first decimal reduction (time required in days to inactivate 90% of the pathogens) to occur. The β is the shape parameter for the curve (β > 1: convex, inactivation increases with time; β < 1: concave, inactivation decreases with time; β = 1: constant inactivation), which describes how the inactivation rate changes over time. These models were selected as these are commonly used in the previous literature, which could allow a more straightforward comparison of the results in this study with previous and future studies. An empirical approach was used to assess model fit by evaluating the root mean square error (RMSE) and the adjusted-R^2^ values of the inactivation models.

### 2.5. Statistical Analysis

This study was conducted in a (2 × 7) + 1 factorial design with 2 inoculum levels and 7 treatments (including PC) plus the NC treatment (n = 15 treatments). The experiments conducted were limited to two independent replications (production day) due to resource and material availability constraints in conducting this study. A two-way analysis of variance (ANOVA) was used to determine the effects of storage time and acidulant treatment on *Salmonella* survival in the treated RMBDs. Analysis of variance (ANOVA) with Tukey’s HSD (*p* < 0.05) was used to determine the effects of inoculation level and acidulant treatment on the survival of *Salmonella* in RMBD patties during refrigerated storage based on the log reductions calculated and model fitting results. In all responses measured or calculated, replication was considered as a random effect during the ANOVA analysis. All statistical analyses were done using JMP Pro version 16.0 statistical software (SAS Institute, Inc., Cary, NC, USA).

## 3. Results

### 3.1. Survival of Salmonella in RMBDs Treated with Food Acidulants

The negative control (NC) yielded no countable background *Salmonella* present; hence, the NC results were not further shown in the results shown in the manuscript. The inoculation method used in this study resulted in patty inoculation levels of 6.02 ± 0.1 and 9.04 ± 0.2 log CFU/patty for the target low and high inoculation levels, respectively. Table 2 shows the *Salmonella* survival in the treated RMBD during storage. In general, the acidulant inclusion (individual or combined) resulted in lower (*p* < 0.05) *Salmonella* load present in the RMBDs compared to the positive control (PC) across all sampling days during storage. At 6 log inoculation level, the addition of combined acidulants (LE + CR, LE + CE, CE + LR, and LR + CR) was more effective in reducing *Salmonella* load compared to the individual treatments (LE and CE). The lowest *Salmonella* loads were recovered in the patties treated with LE + CR and CE + LR. Conversely, the CE treatment was the least effective among the acidulant treatments used in reducing *Salmonella*. At 9 log inoculation level, similar trends were observed such that the inclusion of acidulants resulted in lower (*p* < 0.05) *Salmonella* loads compared to the PC treatment. The inclusion of LE + CR, CE + LR, and LE + CE acidulants resulted in the lowest *Salmonella* load recovered from the RMBD patties. In addition, the CE treatment was also the least effective against *Salmonella* compared to the other acidulants used. The ANOVA results showed that storage time (*p* < 0.05), treatment (*p* < 0.05), and their interaction (*p* < 0.0001) significantly affected *Salmonella* survival in RMBD patties during refrigerated storage. For all treatments, *Salmonella* survival decreased with longer storage time, and the extent of reduction was affected by the acidulant treatments used at both inoculation levels.

The total reduction in *Salmonella* load observed in the RMBD patties at the end of storage (22 days) is shown in Table 3. Among the treatments, the lowest reduction (0.70 and 0.91 logs) was observed for the PC treatment at both inoculation levels. At low inoculation level, the LE + CR inclusion produced the highest reduction (3.51 logs), while the lowest reductions (2.45 and 2.72 logs) were produced from the inclusion of individual acidulants (LE and CE). At high inoculation levels, the use of the combined acidulants (LE + CR, CE + LR, and LE + CE) were the most effective in reducing *Salmonella* in the RMBD patties during storage (3.87 to 4.05 logs). The lowest reductions among acidulant treatments were observed from the RMBD patties treated with LE, CE, and LR + CR treatments. At both inoculation levels, the use of combined acidulants (encapsulated and raw) typically produced higher *Salmonella* reductions in the RMBD compared to using individual encapsulated acidulants (LE and CE). Lastly, the results also indicate that higher initial *Salmonella* load in the RMBD results in higher reductions for all treatments used. 

### 3.2. Inactivation Kinetics of Salmonella in Acidulant-Treated RMBDs

**Figure 1:**
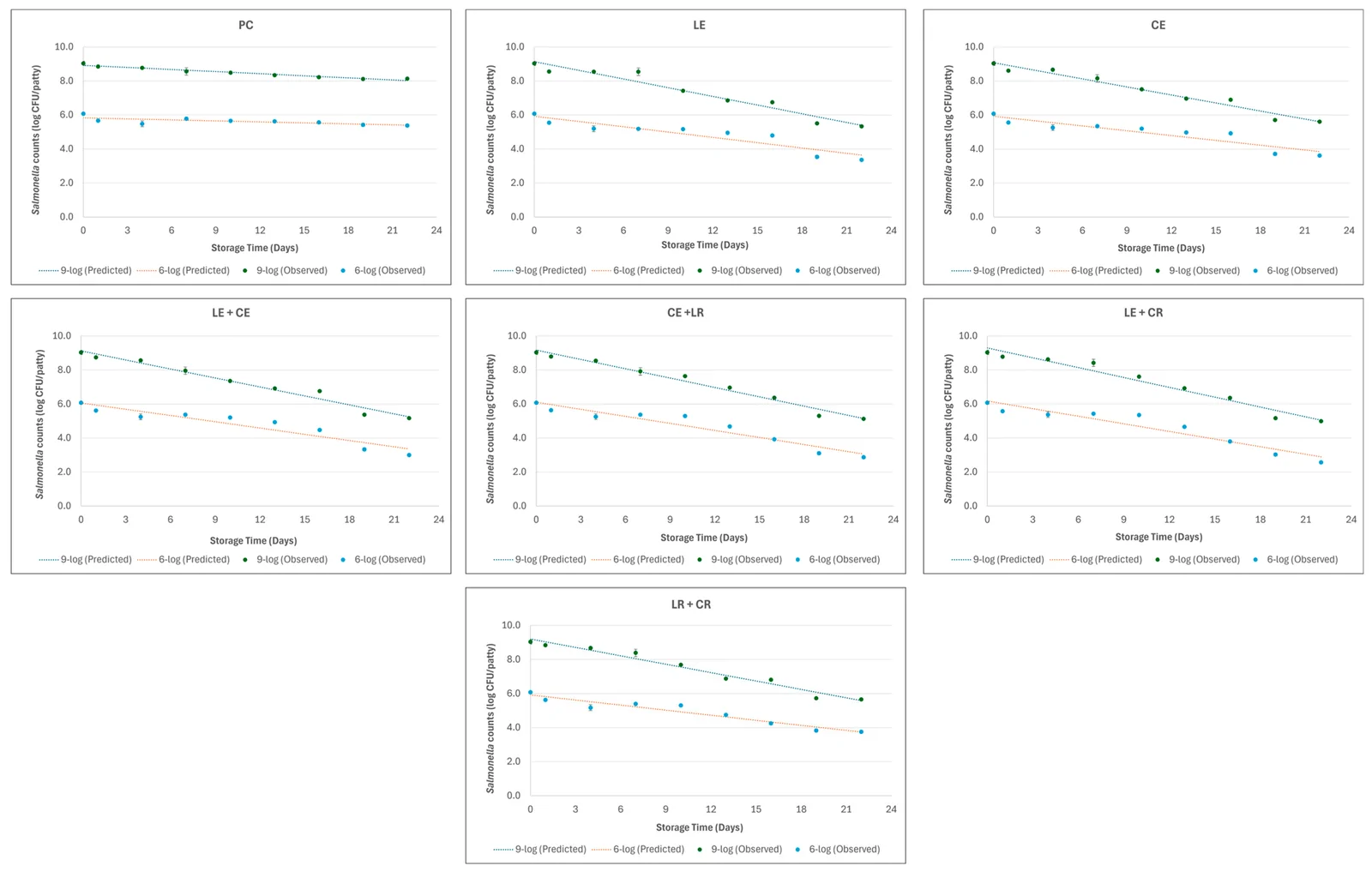
*Salmonella* inactivation curves (under refrigerated storage) in acidulant-treated RMBD patties at both inoculation levels used (9 log and 6 log CFU/patty) using the log-linear model; mean values were used for plotting the trends included. PC—Positive control, LE—encapsulated lactic acid, LR—raw lactic acid, CE—encapsulated citric acid, and CR—raw citric acid.

**Figure 2:**
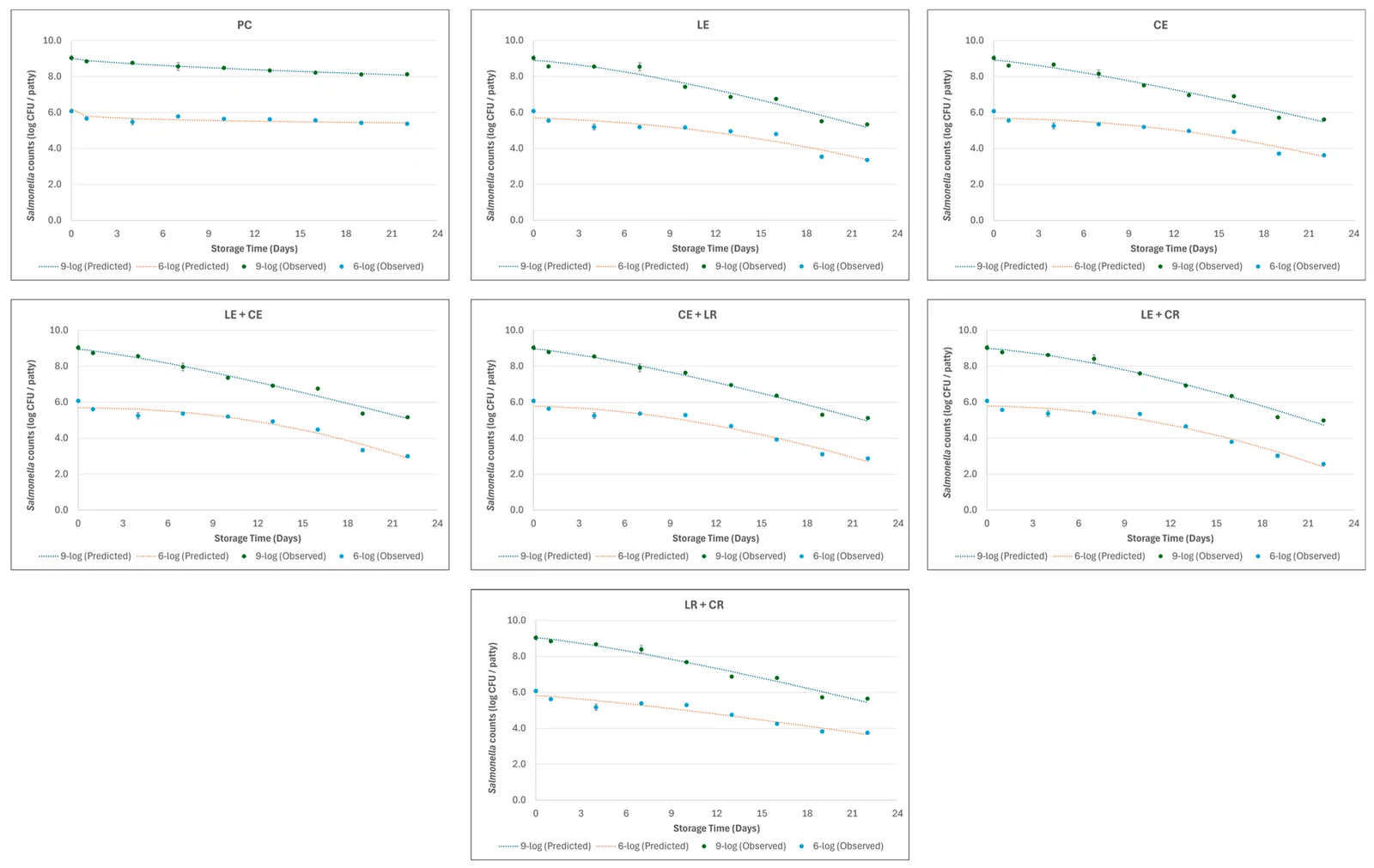
*Salmonella* inactivation curves (under refrigerated storage) in acidulant-treated RMBD patties at both inoculation levels used (9 log and 6 log CFU/patty) using the Weibull model; mean values were used for plotting the trends included. PC—Positive control, LE—encapsulated lactic acid, LR—raw lactic acid, CE—encapsulated citric acid, and CR—raw citric acid.

Aside from the final reductions achieved after storage, the inactivation kinetics of *Salmonella* in RMBDs during storage were also established. The log-linear and Weibull models were selected for this study as these have been more frequently used (i.e., more established) and simpler to implement (i.e., less model coefficients) while still allowing for both non-linear and linear inactivation fitting. The model coefficients calculated at both inoculation levels are shown in Table 4 (log-linear) and Table 5 (Weibull). The predicted values from both models were also calculated based on the model coefficients obtained and are shown in Figure 1 (log-linear) and Figure 2 (Weibull). The fit parameters RMSE (difference between predicted and actual value) and adjusted R^2^ (proportion of variance in dependent variables explained by independent variables) were calculated based on the predicted and actual values.

Both models used (log-linear and Weibull) were significant (*p* < 0.0001; F-value = 139.25 to 409.8) in describing *Salmonella* inactivation at both inoculum levels during storage. The RMSE calculated from the fitted models ranged from 0.16 to 0.55 (log-linear) and 0.10 to 0.34 (Weibull). The calculated adjusted-R^2^ values for the fitted models ranged from 0.43 to 0.97 (log-linear) and 0.46 to 0.98 (Weibull). The model fit results show that both inactivation models used fit well in describing *Salmonella* inactivation due to acidulant addition in refrigerated RMBD. However, the Weibull model performed better based on the lower RMSE values obtained for each treatment. The lower values indicate that the predicted values from the Weibull model are generally closer to the actual values observed from the experiment. Its better fit can also be observed visually (Figure 1 and Figure 2), where the predicted values from the Weibull model are generally closer to the actual values obtained from the experiment. The model fit parameters were also affected by the inoculation level used such that better fit (lower RMSE and higher adjusted R^2^) was observed at high initial *Salmonella* load compared to the lower inoculation level used.

The log-linear model (Table 4) describes a linear inactivation rate for *Salmonella* in the treated RMBD. The inactivation rate is mainly described by the D-value (days needed to reduce *Salmonella* by 1 log cycle), which is calculated from the k_max_ (maximum inactivation rate) value. The ANOVA showed that inoculation level, acidulant treatment, and their interaction significantly affected (*p* < 0.05) the D-value of *Salmonella* in RMBD patties during refrigerated storage. In general, the trends indicated that higher initial inoculation level and the inclusion of acidulants resulted in faster *Salmonella* inactivation (i.e., lower D-value) relative to the control treatment (PC). At 6-log inoculation level, the inclusion of LE + CR resulted in the lowest D-value (2.89 days) compared to using individual and combined similar types of acidulant treatments (LE, CE, LE + CE, and LR + CR). At high inoculation level, the use of LE + CR resulted in the lowest D-value (2.25 days) compared to the other acidulant treatments (LE, CE, LE + CE, CE + LR, and LR + CR). The results also show that higher initial *Salmonella* load contributes to lower D-values, indicating that faster inactivation occurs when higher contamination levels are present.

The Weibull model (Table 5) accounts for the non-linear inactivation of *Salmonella* using the δ and β parameters. The δ indicates the time (days) needed for the first decimal/1 log cycle (90%) reduction in the *Salmonella* load of RMBDs during storage. The β value describes the shape of the non-linear inactivation curve and indicates whether inactivation rate is increasing, decreasing, or constant [24]. The ANOVA analysis showed that inoculation level, acidulant treatment, and their interaction also affected (*p* < 0.05) the inactivation parameters obtained from the Weibull model. At low inoculation level, the inclusion of the combined raw and encapsulated acidulants (CE + LR, LE + CR, and LR + CR) resulted in the lowest (*p* < 0.05) δ values (11.56 to 11.73 days) compared to treatments comprising individual (LE and CE) and combined (LE + CE) acidulants. At high inoculation level, the LE + CE, CE + LR, and LR + CR treatments produced the lowest (*p* < 0.05) δ values (7.36 to 7.77 days) compared to the rest of acidulant treatments added. As for the inoculation level effects, a similar trend was observed such that lower δ values (faster inactivation) were observed with higher initial *Salmonella* load. The shape (β) parameter obtained for the acidulant treatments at both inoculation levels were all > 1 (1.22 to 2.08) indicating that inactivation curves were convex-shaped. This means that *Salmonella* inactivation in the acidulant-treated RMBD patties increased as refrigerated storage time progressed. This shape can be attributed to the diffusion of the added acidulants throughout the RMBD matrix during storage causing an increase in *Salmonella* inactivation as more cells get exposed to the added acids. Conversely, the PC treatment at both inoculation levels had β values < 1 (0.21–0.65) indicating that the *Salmonella* inactivation became concave-shaped. This indicated that *Salmonella* inactivation in the RMBD patties decreased as refrigerated storage progressed. This can be attributed to the eventual adaptation and equilibration of *Salmonella* cells to the RMBD matrix and environmental conditions, which likely slowed down the inactivation.

Overall, both inactivation models show the benefits of acidulant inclusion in reducing *Salmonella* contamination in refrigerated RMBD patties. In both cases, the inclusion of acidulants results in faster inactivation (lower D-value and δ values). The shape parameters obtained from the Weibull model also showed the benefit of acidulant inclusion as the inactivation curve followed a convex shape, indicating increasing *Salmonella* inactivation over time. This change in the inactivation shape indicates that the acidulant inclusion both inhibits *Salmonella* growth and survival in the refrigerated RMBD matrix causing more reductions as storage time progresses.

## 4. Discussion

### 4.1. Survival of Salmonella in Acidulant-Treated RMBDs During Refrigerated Storage

The results from the experiment indicate the benefits of including acidulants in refrigerated RMBD formulation for mitigating *Salmonella* contamination. All the acidulant treatments were limited to a total of 1.0% *w*/*w* inclusion rate as this inclusion rate was established to cause minimal adverse effects on RMBD patty quality indicators such as texture, color, syneresis, and weeping [1]. The results gathered from this study generally agreed with the previous study by Kiprotich et al. [1], which also showed significant reductions in *Salmonella* load during refrigerated storage of acidulant-treated RMBD patties. In general, the use of combined raw and encapsulated acidulants (LE + CR, CE + LR, LE + CE, and LR + CR) resulted in improved efficacy against *Salmonella* compared to using individual acidulants (LE and CE). This trend supports the study hypothesis that combined treatments would increase the efficacy of the acidulant treatment against *Salmonella* by virtue of additive, potentiation, and/or synergistic effects. The observed reductions can be attributed to the more acidic environment produced by the acidulant inclusion in the RMBD patties making it less favorable for *Salmonella* survival and growth. In general, the anti-microbial activity of organic acids is related to their ability to release protons (H^+^) into the bacterial environment (e.g., RMBD); these protons cross the bacterial membrane and lower the internal cellular pH. This acidification effect could then lead to varying anti-microbial effects such as damaging the bacterial membrane and disruption of internal cellular processes leading to bacterial inactivation. From a previous study, acidulant inclusion resulted in a pH reduction from 6.5 to 5.0, making the RMBD patties a less favorable environment for *Salmonella* survival [18]. A higher λ value (109) for *Salmonella* when grown in raw chicken at pH 5 relative to pH 6 and 7 (λ = 63 to 85) indicates that acidic pH inhibits *Salmonella* growth during refrigerated storage [25]. In a separate study, Petrin et al. [26] reported that acidic pH also limits *Salmonella* biofilm formation, which is another important survival function used by enteric pathogens. *Salmonella* inhibition via organic acids is typically facilitated by the anti-microbial effects of acidic pH including alterations in intracellular metabolic functions such as protein phosphorylation, flagellar synthesis, and nutrient transport [27]. Acidic pH also induces physiological and morphological damage to bacterial cells including greater cell protein leakage and cytoplasm membrane disruption [28]. Inherent acid characteristics also influence their anti-microbial efficacy, which explains the differences in the efficacy of both types (raw and encapsulated) of citric and lactic acid tested. Both citric and lactic acid remain neutral and undissociated at low-pH conditions, which allow them to freely cross the bacterial cell membrane, dissociate into anions and cations in the cytoplasm, and cause acidification that leads to structural and functional damage to the bacterial cell [29,30,31]. The *Salmonella* reductions achieved by the combined acid treatments indicate that their relationship is likely additive as slight improvements in the reductions and inactivation rates were observed compared to the individual acid treatments. The results also showed that the acid form (encapsulated and raw) also affected their efficacy against *Salmonella*. For instance, the LR + CR treatment (both raw) resulted in a reduced anti-microbial efficacy relative to the treatments consisting of raw and encapsulated forms. This is likely due to the encapsulation process, which protects the acids from immediate neutralization upon contact caused by the buffering ability of meat proteins in the RMBD matrix [28,32]. Instead, a delayed release occurs for the acids, which allows them to exert more anti-microbial effects when added. This agreed with the findings reported by Kiprotich et al. [1], wherein LR (raw lactic acid) had higher efficacy against *Salmonella* during in vitro testing compared to testing in a raw meat matrix. This limitation was addressed using the combined encapsulated acids (LE + CE), wherein the results showed improved reductions in *Salmonella* relative to the combined raw (LR + CR) treatment. Overall, this also supports the hypothesis of this study that encapsulation improves anti-microbial efficacy of acids as it induces a delayed release of acids instead of being immediately neutralized by meat proteins.

The results of the study also showed the importance of *Salmonella* inoculation level on the efficacy of the acidulants added in the RMBD patties. In general, higher inoculation levels resulted in better reductions and faster inactivation of *Salmonella* in the stored RMBD with added acidulants. This trend could be due to the “inoculum effect”, wherein the population size affects the rate of microbial inactivation via quorum sensing [33]. In this mechanism, bacteria can regulate their population size via signal molecules that help initiate stress responses that aid in bacterial survival [34]. For instance, once *Salmonella* Typhimurium reaches maximum population density, chemical signals responsible for growth and metabolic activity get limited to allow the microbial population to thrive within the available resources in their environment [35]. Hence, future studies are needed to establish the potential effects of quorum sensing that influence *Salmonella* survival in RMBDs with added acids. In addition, the higher inoculation level likely increases the chance of the diffused acidulants interacting with the *Salmonella* cells facilitating the higher inactivation observed.

### 4.2. Inactivation Kinetics of Salmonella in RMBDs During Refrigerated Storage

This study used inactivation models to allow the predictive quantification of a “hurdle concept” assessing the cumulative effects of inoculum size, storage time, and acidulant treatment, which can help support food safety decisions in handling high-risk food products [33]. The log-linear model uses a simpler approach to describing *Salmonella* inactivation kinetics in refrigerated RMBDs as it assumes similar resistance for the entire microbial population against an anti-microbial treatment [21,36]. The Weibull model accounts for varying resistances (β value) in the microbial population against anti-microbial treatment and describes a statistical model of the distribution of the microbial inactivation of the target microorganism [23].

The established inactivation kinetics for *Salmonella* during refrigerated storage of RMBDs showed that adding acidulants increased the rate of *Salmonella* inactivation (lower D-value and δ value). The reduction was amplified when combined acidulant treatments (LE + CE, CE + LR, LE + CR, and LR + CR) were used. The combined use of raw and encapsulated acidulants also resulted in improved inactivation rates, which supported the higher reductions observed. Comparing both models, the Weibull model was better suited for describing the *Salmonella* inactivation in the RMBD patties based on the lower RMSE values observed across all treatments. This is due to the ability of the Weibull model to account for potential changes in the rate of inactivation of the *Salmonella* population across time [19]. In this study, the *Salmonella* inactivation curve was convex-shaped (β > 1), which indicated increasing reductions across storage time [37]. This effect is attributed to the use of encapsulated acids allowing for a delayed release of acidulants into the meat matrix. Hence, the slower initial reductions can be linked to the need for the acids released to build up into a critical concentration in the RMBD matrix to facilitate *Salmonella* inactivation. In contrast to this, the PC treatment had a concave shape, indicating a reducing inactivation rate. This is likely due to the increasing adaptation of *Salmonella* in the RMBD matrix and refrigerated conditions. Overall, the results demonstrate the importance of organic acids as antimicrobial ingredients in RMBD production. These findings are crucial as this pet food category uses raw ingredients that are prone to pathogen contamination [38]. 

## 5. Conclusions

This study demonstrated the anti-microbial effects of encapsulated organic acids against *Salmonella* in RMBDs. The use of individual encapsulated acids resulted in a maximum reduction ranging from 2.7 to 3.7 logs depending on the initial *Salmonella* level present. These reductions were improved by combining both raw and encapsulated acid forms as the maximum reductions increased to 3.5 to 4.1 logs at the end of the storage period. In both cases, higher contamination levels resulted in higher reductions observed. *Salmonella* inactivation in the acid-treated RMBD patties was better described by the Weibull model as inactivation increased over time under refrigeration. Overall, including both raw and encapsulated acids in RMBD formulations was a viable approach for mitigating *Salmonella* contamination, and that the combined usage of raw and encapsulated acids could further increase its efficacy. In addition, the results indicated that the acidulants worked by both reducing the existing *Salmonella* contamination present and preventing growth during refrigerated storage of RMBDs.

This study contributes to the knowledge base on using organic acids as an anti-microbial measure against foodborne pathogens such as *Salmonella* in RMBDs. Although the results were promising, several limitations still need to be addressed for fully validating encapsulated organic acids as an anti-microbial ingredient for RMBDs. The major limitations encountered in this study were the use of only one meat type, few replications, and use of a single storage condition (4 °C). Hence, future studies could include (1) using more biological replicates for improving statistical power, (2) assessing effects of RMBD formulation differences and storage temperature variations on the efficacy of encapsulated and raw organic acids against *Salmonella*, (3) use of recovery methods for sub-lethally injured *Salmonella* cells, (4) conducting validation studies at larger production scale using more natural levels of *Salmonella* present, and (5) fully assessing the effects of acidulant inclusion on the quality characteristics and palatability of RMBDs to companion animals.

## Figures and Tables

**Table 1:** Treatments used for assessing the effects of food acidulants on *Salmonella* survival in RMBDs.

Inoculation Level (log CFU/Patty)	Treatments	Concentration of Acidulants (%*w*/*w*)
9 (High)	NC	None
	PC	None
	LE	1.0%
	CE	1.0%
	LE + CE	0.5% LE + 0.5% CE
	CE + LR	0.5% CE + 0.5% LR
	LE + CR	0.5% LE + 0.5% CR
	LR + CR	0.5% LR + 0.5% CR
6 (Low)	NC	None
	PC	None
	LE	1.0%
	CE	1.0%
	LE + CE	0.5% LE + 0.5% CE
	CE + LR	0.5% CE + 0.5% LR
	LE + CR	0.5% LE + 0.5% CR
	LR + CR	0.5% LR + 0.5% CR

PC—Positive control, NC—negative control, LE—encapsulated lactic acid, LR—raw lactic acid, CE—encapsulated citric acid, and CR—raw citric acid.

**Table 2:** *Salmonella* counts (mean ± SD) in RMBD patties treated with food acidulants during refrigerated storage (4 °C, 22 days).

Treatments	*Salmonella* Counts (log CFU/Patty)								
	Storage Time (Days)								
	0	1	4	7	10	13	16	19	22
6 log CFU/patty (Low)									
PC	6.08 (0.9) aA	5.67 (0.05) aB	5.48 (0.02) aD	5.79 (0.06) aC	5.65 (0.03) aD	5.62 (0.02) aE	5.57 (0.03) aF	5.42 (0.07) aG	5.38 (0.03) aH
LE	6.08 (0.9) cA	5.55 (0.08) cB	5.19 (0.09) cD	5.18 (0.15) cC	5.16 (0.09) cD	4.95 (0.03) cE	4.80 (0.07) cF	3.54 (0.03) cG	3.36 (0.02) cH
CE	6.08 (0.9) bA	5.56 (0.1) bB	5.27 (0.12) bD	5.35 (0.03) bC	5.20 (0.06) bD	4.98 (0.01) bE	4.93 (0.02) bF	3.72 (0.03) bG	3.62 (0.03) bH
LE + CE	6.08 (0.9) dA	5.62 (0.07) dB	5.25 (0.06) dD	5.37 (0.0) dC	5.21 (0.04) dD	4.93 (0.03) dE	4.48 (0.03) dF	3.34 (0.05) dG	3.00 (0.11) dH
CE + LR	6.08 (0.9) eA	5.64 (0.06) eB	5.26 (0.17) eD	5.36 (0.05) eC	5.29 (0.09) eD	4.68 (0.04) eE	3.93 (0.06) eF	3.11 (0.09) eG	2.87 (0.07) eH
LE + CR	6.08 (0.9)eA	5.58 (0.09) eB	5.37 (0.15) eD	5.43 (0.06) eC	5.35 (0.03) eD	4.66 (0.06) eE	3.80 (0.07) eF	3.03 (0.07) eG	2.57 (0.11) eH
LR + CR	6.08 (0.9) cA	5.63 (0.06) cB	5.17 (0.09) cD	5.39 (0.02) cC	5.30 (0.03) cD	4.75 (0.03) cE	4.25 (0.10) cF	3.83 (0.03) cG	3.76 (0.03) cH
9 log CFU/patty (High)									
PC	9.04 (0.13) aA	8.85 (0.06) aB	8.77 (0.06) aC	8.56 (0.06) aD	8.49 (0.04) aE	8.34 (0.08) aF	8.22 (0.07) aG	8.12 (0.07) aH	8.13 (0.18) aI
LE	9.04 (0.13) dA	8.56 (0.11) dB	8.55 (0.10) dC	8.54 (0.09) dD	7.42 (0.06) dE	6.86 (0.03) dF	6.75 (0.05) dG	5.51 (0.05) dH	5.34 (0.03) dI
CE	9.04 (0.13) cA	8.16 (0.17) cB	8.67 (0.03) cC	8.16 (0.12) cD	7.51 (0.04) cE	6.97 (0.02) cF	6.90 (0.04) cG	5.71 (0.05) cH	5.61 (0.02) cI
LE + CE	9.04 (0.13) eA	8.74 (0.03) eB	8.57 (0.06) eC	7.96 (0.12) eD	7.36 (0.04) eE	6.92 (0.02) eF	6.76 (0.06) eG	5.37 (0.03) eH	5.17 (0.09) eI
CE + LR	9.04 (0.13) eA	8.80 (0.06) eB	8.55 (0.04) eC	7.92 (0.22) eD	7.64 (0.02) eE	6.96 (0.02) eF	6.37 (0.05) eG	5.31 (0.05) eH	5.13 (0.06) eI
LE + CR	9.04 (0.13) eA	8.78 (0.09) eB	8.63 (0.05) eC	8.42 (0.14) eD	7.60 (0.02) eE	6.93 (0.02) eF	6.35 (0.06) eG	5.17 (0.02) eH	4.99 (0.06) eI
LR + CR	9.04 (0.13) bA	8.85 (0.02) bB	8.68 (0.03) bC	8.39 (0.09) bD	7.69 (0.01) bE	6.88 (0.02) bF	6.81 (0.02) bG	5.72 (0.02) bH	5.65 (0.03) bI

Values enclosed in parenthesis are standard deviations (SDs); mean counts with different uppercase letters in each treatment (row) are significantly different due to storage time (*p* < 0.05); mean counts with different lowercase letters in each storage time (column) are significantly different due to treatment (*p* < 0.05). PC—Positive control, LE—encapsulated lactic acid, LR—raw lactic acid, CE—encapsulated citric acid, and CR—raw citric acid.

**Table 3:** Total *Salmonella* reductions observed in the RMBD patties treated with acidulants after refrigerated storage (4 °C, 22 days).

Treatment	*Salmonella* Reduction After Storage	
	6 log CFU/Patty (Low)	9 log CFU/Patty (High)
PC	0.70 (0.09) eB	0.91 (0.26) dA
LE	2.72 (0.10) cB	3.70 (0.15) bCA
CE	2.45 (0.07) dCB	3.42 (0.12) cA
LE + CE	3.08 (0.17) bB	3.87 (0.13) bA
CE + LR	3.21 (0.16) bB	3.91 (0.08) aA
LE + CR	3.51 (0.18) aB	4.05 (0.19) aA
LR + CR	2.32 (0.11) dB	3.38 (0.15) cA

Reductions were calculated as the difference in *Salmonella* counts of the RMBD patties at day 0 and 22; values enclosed in parenthesis are standard deviations (SDs); means with different lowercase letters in each column are significantly different due to treatment (*p* < 0.05); means with different uppercase letters in each row are significantly different due to inoculation level (*p* < 0.05). PC—Positive control, LE—encapsulated lactic acid, LR—raw lactic acid, CE—encapsulated citric acid, and CR—raw citric acid.

**Table 4:** Log-linear model fitting results for the *Salmonella* counts obtained in the RMBD patties treated with acidulants during refrigerated storage (4 °C, 22 days).

Treatments	Model Parameters		Goodness of Fit Indices	
	k_max_	D-Value (Days)	RMSE	Adjusted R-Square
6 log CFU/patty (Low)				
PC	0.045 (0.01)	23.75 (7.25) aA	0.16	0.43
LE	0.24 (0.01)	4.22 (0.17) bA	0.37	0.82
CE	0.21 (0.01)	4.60 (0.11) bA	0.34	0.82
LE + CE	0.28 (0.01)	3.54 (0.12) cA	0.38	0.86
CE + LR	0.32 (0.01)	3.15 (0.05) cA	0.36	0.90
LE + CR	0.35 (0.01)	2.89 (0.05) dA	0.55	0.88
LR + CR	0.23 (0.01)	4.35 (0.01) bA	0.25	0.91
9 log CFU/patty (High)				
PC	0.09 (0.01)	10.90 (1.18) aB	0.10	0.89
LE	0.39 (0.01)	2.55 (0.03) bB	0.34	0.94
CE	0.36 (0.01)	2.76 (0.07) bB	0.26	0.95
LE + CE	0.41 (0.01)	2.45 (0.06) cB	0.26	0.97
CE + LR	0.42 (0.01)	2.38 (0.05) dB	0.24	0.97
LE + CR	0.45 (0.02)	2.25 (0.09) eB	0.32	0.96
LR + CR	0.38 (0.01)	2.63 (0.06) bB	0.25	0.96

k_max_ and D-values are coefficients obtained from fitting the model; values enclosed in parenthesis are standard deviations (SDs); mean values with different lowercase letters for each inoculation level are significantly different (*p* < 0.05) due to treatment; mean values with different uppercase letters for each treatment are significantly different due to inoculation level. PC—Positive control, LE—encapsulated lactic acid, LR—raw lactic acid, CE—encapsulated citric acid, and CR—raw citric acid; RMSE—root mean square error.

**Table 5:** Weibull model fitting results for the *Salmonella* counts obtained in the RMBD patties treated with acidulants during refrigerated storage (4 °C, 22 days).

Treatments	Model Parameters		Goodness of Fit Indices	
	δ (Days)	β	RMSE	Adjusted R-Square
6 log CFU/patty (Low)				
PC	33.52 (5.34) aA	0.21 (0.03)	0.17	0.46
LE	14.65 (0.27) bA	2.08 (0.12)	0.34	0.86
CE	15.00 (0.69) bA	1.98 (0.21)	0.32	0.85
LE + CE	13.64 (0.11) cA	2.15 (0.09)	0.28	0.92
CE + LR	11.56 (0.19) dA	1.75 (0.09)	0.29	0.94
LE + CR	11.73 (0.17) cdA	1.94 (0.08)	0.27	0.95
LR + CR	11.63 (0.70) dA	1.22 (0.11)	0.26	0.90
9 log CFU/patty (High)				
PC	23.99 (4.58) aB	0.65 (0.13)	0.10	0.91
LE	8.37 (0.28) bB	1.37 (0.04)	0.32	0.95
CE	7.96 (0.82) bB	1.23 (0.01)	0.25	0.96
LE + CE	7.34 (0.24) cB	1.23 (0.03)	0.25	0.97
CE + LR	7.36 (0.89) dB	1.27 (0.13)	0.19	0.98
LE + CR	8.00 (0.33) cdB	1.43 (0.03)	0.23	0.98
LR + CR	7.77 (0.40) dB	1.23 (0.05)	0.24	0.97

δ and β are coefficients obtained from fitting the model; values enclosed in parenthesis are standard deviations (SDs); mean values with different lowercase letters for each inoculation level are significantly different (*p* < 0.05) due to treatment; mean values with different uppercase letters for each treatment are significantly different due to inoculation level. PC—Positive control, LE—encapsulated lactic acid, LR—raw lactic acid, CE—encapsulated citric acid, and CR—raw citric acid; RMSE—root mean square error.

## Data Availability

The data supporting the findings from this study are available by reasonable request to the corresponding authors due to commercial restrictions.

## References

[1] Kiprotich S., Altom E., Mason R., Trinetta V., Aldrich C.G. (2023). Application of encapsulated and dry-plated food acidulants to control *Salmonella enterica* in raw meat-based diets for dogs. J. Food Prot..

[2] Raditic D.M. (2021). Insights into commercial pet foods. Vet. Clin. Small Anim. Pract..

[3] Delgado-Andrade C., Tessier F.J., Niquet-Leridon C., Seiquer I., Navarro M.P. (2012). Study of the urinary and faecal excretion of N ε-carboxymethyllysine in young human volunteers. Amino Acids.

[4] van Rooijen C., Bosch G., van der Poel A.F., Wierenga P.A., Alexander L., Hendriks W.H. (2014). Quantitation of Maillard reaction products in commercially available pet foods. J. Agric. Food Chem..

[5] Kiprotich S.S., Aldrich C.G. (2022). A review of food additives to control the proliferation and transmission of pathogenic microorganisms with emphasis on applications to raw meat-based diets for companion animals. Front. Vet. Sci..

[6] Market Research Future (2025). Global Raw Pet Food Market Overview. https://www.marketresearchfuture.com/reports/raw-pet-food-market-23005.

[7] Association of American Feed Control Officials (AAFCO) (2020). Official Publication.

[8] Freeman L.M., Chandler M.L., Hamper B.A., Weeth L.P. (2013). Current knowledge about the risks and benefits of raw meat–based diets for dogs and cats. J. Am. Vet. Med. Assoc..

[9] Acuff H.L., Dainton A.N., Dhakal J., Kiprotich S., Aldrich C.G. (2021). Sustainability and Pet Food: Is There a Role for Veterinarians?. Vet. Clin. N. Am. Small Anim..

[10] Solís D., Toro M., Navarrete P., Faúndez P., Reyes-Jara A. (2022). Microbiological quality and presence of foodborne pathogens in raw and extruded canine diets and canine fecal samples. Front. Vet. Sci..

[11] Ramos C.P., Kamei C.Y.I., Viegas F.M., de Melo Barbieri J., Cunha J.L.R., Hounmanou Y.M.G., Coura F.M., Santana J.A., Lobato F.C.F., Bojesen A.M. (2022). Fecal shedding of multidrug resistant *Escherichia coli* isolates in dogs fed with raw meat-based diets in Brazil. Antibiotics.

[12] Viegas F.M., Ramos C.P., Xavier R.G.C., Lopes E.O., Júnior C.A.O., Bagno R.M., Diniz A.N., Lobato F.C.F., Silva R.O.S. (2020). Fecal shedding of *Salmonella* spp., *Clostridium perfringens*, and *Clostridioides difficile* in dogs fed raw meat-based diets in Brazil and their owners’ motivation. PLoS ONE.

[13] Morelli G., Bastianello S., Catellani P., Ricci R. (2019). Raw meat-based diets for dogs: Survey of owners’ motivations, attitudes and practices. BMC Vet. Res..

[14] Empert-Gallegos A., Hill S., Yam P.S. (2020). Insights into dog owner perspectives on risks, benefits, and nutritional value of raw diets compared to commercial cooked diets. PeerJ.

[15] Food and Drug Administration—FDA (2017). FSMA Final Rule for Preventive Controls for Animal Food. https://www.fda.gov/food/food-safety-modernization-act-fsma/fsma-final-rule-preventive-controls-animal-food.

[16] Serra-Castelló C., Possas A., Jofré A., Garriga M., Bover-Cid S. (2022). Enhanced high hydrostatic pressure lethality in acidulated raw pet food formulations was pathogen species and strain dependent. Food Microbiol..

[17] Sheen S., Cassidy J., Scullen B., Uknalis J., Sommers C. (2015). Inactivation of *Salmonella* spp. in ground chicken using high pressure processing. Food Control.

[18] Kiprotich S., Altom E., Mason R., Aldrich C.G. (2021). Application of encapsulated lactic acid to control the growth and multiplication of *Salmonella enterica* in raw meat-based diets for dogs. Kans. Agric. Exp. Stn. Res. Rep..

[19] Buzrul S. (2022). The Weibull model for microbial inactivation. Food Eng. Rev..

[20] de Oliveira G.M.C. (2019). Modeling Microbial Inactivation Subjected to Nonisothermal and Non-Thermal Food Processing Technologies. Ph.D. Thesis.

[21] Geeraerd A.H., Valdramidis V.P., Van Impe J.F. (2005). GInaFiT, a freeware tool to assess non-log-linear microbial survivor curves. Int. J. Food Microbiol..

[22] Geeraerd A., Van Impe J. (2007). GInaFiT. Revealing the time-dependence of microbial survival under food processing, food preservation or environmental stress conditions. 5th International Conference Predictive Modelling in Foods-Conference Proceedings.

[23] Mafart P., Couvert O., Gaillard S., Leguérinel I. (2002). On calculating sterility in thermal preservation methods: Application of the Weibull frequency distribution model. Int. J. Food Microbiol..

[24] van Boekel M.A. (2002). On the use of the Weibull model to describe thermal inactivation of microbial vegetative cells. Int. J. Food Microbiol..

[25] Peruzzolo M., Danielli A.J., Fischer B., Junges A., Colet R., Steffens C., de Souza M.A.S.F., Cansian R.L., Backes G.T. (2024). Growth Effects as a Function of pH and Temperature of *Salmonella enterica* serovar Choleraesuis in in vitro Tests and Chicken Meat: Prediction and Modeling. Indian J. Microbiol..

[26] Petrin S., Mancin M., Losasso C., Deotto S., Olsen J.E., Barco L. (2022). Effect of pH and salinity on the ability of *Salmonella* serotypes to form biofilm. Front. Microbiol..

[27] Lu H.J., Breidt F., Perez-Diaz I.M., Osborne J.A. (2011). Antimicrobial effects of weak acids on the survival of *Escherichia coli* O157:H7 under anaerobic conditions. J. Food Prot..

[28] Wang C., Chang T., Yang H., Cui M. (2015). Antibacterial mechanism of lactic acid on physiological and morphological properties of *Salmonella* Enteritidis, *Escherichia coli* and *Listeria monocytogenes*. Food Control.

[29] Burel C., Kala A., Purevdorj-Gage L. (2021). Impact of pH on citric acid antimicrobial activity against Gram-negative bacteria. Lett. Appl. Microbiol..

[30] Kundukad B., Udayakumar G., Grela E., Kaur D., Rice S.A., Kjelleberg S., Doyle P.S. (2020). Weak acids as an alternative anti-microbial therapy. Biofilm.

[31] Mani-López E., García H.S., López-Malo A. (2012). Organic acids as antimicrobials to control *Salmonella* in meat and poultry products. Int. Food Res. J..

[32] Yeh Y., De Moura F.H., Van Den Broek K., De Mello A.S. (2018). Effect of ultraviolet light, organic acids, and bacteriophage on *Salmonella* populations in ground beef. Meat Sci..

[33] Sholpan A., Lamas A., Cepeda A., Franco C.M. (2021). *Salmonella* spp. quorum sensing: An overview from environmental persistence to host cell invasion. AIMS Microbiol..

[34] Bidlas E., Du T., Lambert R.J. (2008). An explanation for the effect of inoculum size on MIC and the growth/no growth interface. Int. J. Food Microbiol..

[35] Surette M.G., Bassler B.L. (1998). Quorum sensing in *Escherichia coli* and *Salmonella* Typhimurium. Proc. Natl. Acad. Sci. USA.

[36] Bevilacqua A., Speranza B., Sinigaglia M., Corbo M.R. (2015). A focus on the death kinetics in predictive microbiology: Benefits and limits of the most important models and some tools dealing with their application in foods. Foods.

[37] Albert I., Mafart P. (2005). A modified Weibull model for bacterial inactivation. Int. J. Food Microbiol..

[38] Bentum K.E., Kuufire E., Nyarku R., Osei V., Price S., Bourassa D., Samuel T., Jackson C.R., Abebe W. (2025). Salmonellosis in Cattle: Sources and Risk of Infection, Control, and Prevention. Zoonotic Dis..

